# Bifidobacteria Prevent Tunicamycin-Induced Endoplasmic Reticulum Stress and Subsequent Barrier Disruption in Human Intestinal Epithelial Caco-2 Monolayers

**DOI:** 10.1371/journal.pone.0162448

**Published:** 2016-09-09

**Authors:** Takuya Akiyama, Kenji Oishi, Andy Wullaert

**Affiliations:** 1 Yakult Honsha European Research Center for Microbiology ESV, Ghent, Belgium; 2 Yakult Central Institute, Tokyo, Japan; 3 Inflammation Research Center, VIB, Ghent, Belgium; 4 Internal Medicine Department, Ghent University, Ghent, Belgium; National Institute for Agronomic Research, FRANCE

## Abstract

Endoplasmic reticulum (ER) stress is caused by accumulation of unfolded and misfolded proteins in the ER, thereby compromising its vital cellular functions in protein production and secretion. Genome wide association studies in humans as well as experimental animal models linked ER stress in intestinal epithelial cells (IECs) with intestinal disorders including inflammatory bowel diseases. However, the mechanisms linking the outcomes of ER stress in IECs to intestinal disease have not been clarified. In this study, we investigated the impact of ER stress on intestinal epithelial barrier function using human colon carcinoma-derived Caco-2 monolayers. Tunicamycin-induced ER stress decreased the trans-epithelial electrical resistance of Caco-2 monolayers, concomitant with loss of cellular plasma membrane integrity. Epithelial barrier disruption in Caco-2 cells after ER stress was not caused by caspase- or RIPK1-dependent cell death but was accompanied by lysosomal rupture and up-regulation of the ER stress markers Grp78, sXBP1 and Chop. Interestingly, several bifidobacteria species inhibited tunicamycin-induced ER stress and thereby diminished barrier disruption in Caco-2 monolayers. Together, these results showed that ER stress compromises the epithelial barrier function of Caco-2 monolayers and demonstrate beneficial impacts of bifidobacteria on ER stress in IECs. Our results identify epithelial barrier loss as a potential link between ER stress and intestinal disease development, and suggest that bifidobacteria could exert beneficial effects on this phenomenon.

## Introduction

Intestinal immune homeostasis is maintained by multiple signalling pathways acting in intestinal epithelial cells (IECs) to preserve epithelial permeability. Defects in intestinal barrier function indeed are associated with various gastrointestinal disorders such as Inflammatory Bowel Disease (IBD), celiac disease, Irritable Bowel Syndrome (IBS) and necrotizing enterocolitis (NEC).[1, 2] Therefore, signalling pathways that aim to preserve the intestinal epithelial barrier are potential therapeutic targets for the prevention or treatment of intestinal inflammation.

One of the increasingly recognized signalling pathways involved in regulating intestinal health is endoplasmic reticulum (ER) stress. ER stress is caused by accumulating misfolded proteins, which induces signalling pathways that collectively initiate the unfolded protein response (UPR) attempting to restore protein folding, increase ER biosynthetic machinery and maintain cellular homeostasis.[3, 4] However, excessive ER stress can lead to failure in protein secretion, cell injury or even cell death,[5] all of which can contribute to disrupting intestinal homeostasis.[6] Indeed, genetic polymorphisms in the UPR transcription factor X-box binding protein (XBP)-1 predisposes to IBD development.[7] In accordance with this potential pathologic role for ER stress in human intestinal diseases, elevated ER stress was detected in IECs from IBD as well as NEC patients.[8–11] These observations in patients suggesting ER stress involvement in intestinal disease pathogenesis were confirmed in multiple experimental mouse models showing a causative role for ER stress in intestinal inflammation.[7, 8, 12–17] In addition, administration of the ER stress inhibitor tauroursodeoxycholic acid (TUDCA) was shown to ameliorate various models of colitis in mice.[13, 15, 18] However, the cellular outcomes of ER stress in human IECs and the underlying mechanisms regulating these effects are not fully understood.

Here we demonstrate that tunicamycin (TM)-induced ER stress disrupts epithelial barrier function in differentiated human colon carcinoma-derived Caco-2 epithelial monolayers. We identify lysosome rupture as a predominant mechanism underlying TM-induced barrier loss in these human IEC monolayers. Moreover, in this study we demonstrate a protective effect of *Bifidobacterium* species on ER stress induced barrier disruption in Caco-2 monolayers, suggesting that particular bacteria are capable of modulating intestinal epithelial ER stress and thus may have beneficial effects in ER-stress associated intestinal inflammation.

## Materials and Methods

### Cell culture and differentiation to epithelial monolayer

Human colon carcinoma Caco-2 cells (obtained from the European collection of cell cultures (ECACC), catalogue number 09042001) were cultured in DMEM-GlutaMAX medium supplemented with 10% (v/v) Foetal Bovine Serum (FBS), 1% Non-essential Amino Acids and 1 mM Sodium Pyruvate (Life Technologies) at 37°C in a humidified 5% (v/v) CO_2_ atmosphere. For differentiating these Caco-2 cells to epithelial monolayers, the Biocoat HTS Caco-2 Assay System (Beckton Dickinson) was used according to the manufacturer’s protocol. Briefly, Caco-2 cells were seeded in fibrillar collagen pre-coated inserts (1 μm pores, surface area 0,3 cm^2^) at a density of 10^5^ cells per insert in DMEM-GlutaMAX medium supplemented as above. On day 3, both apical and basolateral medium was exchanged to Entero-Stim Differentiation Medium supplemented with Mito+ Serum Extender. On day 4, the Entero-Stim Differentiation Medium with Mito+ Serum Extender was refreshed. In case of pre-incubation with Bifidobacteria or Lactobacilli, 4.0×10^8^ heat-killed bacteria per well were added at this point. After 12 hours of incubation (day 5), trans-epithelial electrical resistance (TEER) was measured using an STX100F electrode (World Precision Instruments) to verify the formation of tight monolayers. Wells with a minimum TEER of 1000 Ω/cm^2^ were considered as truly differentiated epithelial monolayers and only those wells were used in the experiments. Values were measured as Ω/cm^2^, and expressed as percent TEER of the identical monolayer as measured just before treatment.

### Chemical treatments on epithelial monolayer

ER stress was induced by adding tunicamycin (TM) (Sigma) at the indicated final concentration to the apical compartment of Caco-2 monolayers. As a control, vehicle (VH, corresponding to a final concentration of 0,1% DMSO) was added to the cells, which did not affect Caco-2 epithelial permeability when compared to non-treated monolayer controls (data not shown). For ER stress inhibition 5 mM of tauroursodeoxycholic acid (TUDCA) was added one hour prior to the TM stimulation. For lysosomal rupture experiments 30 mM of sucrose (Sigma) was added one hour prior to TM stimulation.

### Trypan blue and acridine orange stainings, and DNA fragmentation assay

Caco-2 monolayers were gently rinsed with ice-cold PBS and incubated with 0.2% (v/v) trypan blue solution (Sigma) for 15 minutes at 4°C, or with 10 μg/ml acridine orange (Sigma) for 15 minutes at 37°C. After rinsing with ice-cold PBS, monolayers were detached from the fibrillar collagen pre-coated inserts. For acridine orange staining, monolayers were mounted with Fluoromount (Sigma) and red fluorescence was imaged under a fluorescence microscope with an excitation wavelength of 550 nm. For Trypan blue stainings, the monolayers were mounted with PBS on slides for optical microscope imaging. Trypan blue positive cells in a monolayer were quantified by summarizing the blue area of four fixed independent sections imaged under 100x magnification. The results of three monolayers or more were averaged in a single experiment for each condition. Caspase-dependent DNA fragmentation of Caco-2 monolayers was measured using the Cell death detection ELISA (Roche) based on nucleosome immunoreactivity according to the manufacturer’s instructions.

### mRNA expression analysis

Total RNA from Caco-2 monolayers was isolated by lysing cells with Trizol (Life Technologies) followed by RNA extraction using RNeasy columns (Qiagen). Quality of RNA was assessed by Nanodrop spectrophotometric analysis (Thermo Scientific), and a β-Actin PCR was performed to exclude contamination with genomic DNA using 5’-CATGTACGTTGCTATCCAGGC-3’ and 5’-CTCCTTAATGTCACGCACGAT-3’ primers. Then, 200 ng of RNA was used for cDNA synthesis with the Superscript lll kit (Life Technologies) according to the manufacturer’s instructions. Resulting cDNA was diluted to 5 ng/μl with nuclease-free H_2_O and 2 μl of cDNA solution was subjected to a βActin PCR to assure proper synthesis of cDNA in all samples. Finally, 10 ng of cDNA was used for quantitative real time PCR using TaqMan gene expression assays (Applied Biosystems) in 384-well plates according to the manufacturer’s instructions. Relative mRNA expression levels were determined according to the comparative ΔΔCT method, normalized to the level of the reference gene *Tbp*. The following Taqman gene expression probes were used: Tbp Hs00427621_m1, Chop Hs00358796_g1, Grp78 Hs00607129_gH. The custom designed 5’-CGCAGCAGGTGCAGGCCCA-3’ Taqman probe for spliced Xbp1 was used for a specific Xbp1s assay with sense 5’-GAATGGACACGCTGGATCCT-3’ and antisense 5’-TCAGAATCTGAAGAGGCAACAG-3’ primers as described.[19]

### Immunoblotting

Caco-2 monolayers were lysed directly in Laemmli buffer supplemented with 5% (v/v) 2-mercaptoethanol and boiled for 15 minutes. Equal amounts of lysate were separated by electrophoresis on SDS-polyacrylamide gels (Biorad) under denaturing conditions. Proteins were transferred to PVDF membranes (Millipore) via wet electrotransfer. After protein blotting, membranes were blocked in PBS with 0.1% (v/v) Tween-20 with either 5% (w/v) non-fat dry milk or 5% (w/v) BSA. Primary antibodies against active Caspase-3 (BD Biosciences), CHOP (Cell Signalling), GRP78 (Cell Signalling), XBP1s (BioLegend), or βActin (Santa Cruz) were applied overnight at 4°C. After washing, membranes were incubated with HRP-conjugated secondary antibodies raised against mouse, rabbit or goat immunoglobulins (Abcam). Protein bands were visualized with ECL (Pierce) and ChemiDoc MP System (Bio-Rad). Quantification of the bands were performed by imageJ software, with the intensity values normalized to the corresponding βActin band.

### Preparation of heat-killed bacteria

Overnight cultures of *Lactobacillus casei* Shirota YIT 9029, *L*. *plantarum* YIT 0102^T^, *L*. *rhamnosus* YIT 0105^T^, *L*. *acidophilus* YIT 0070^T^, *Bifidobacterium breve* YIT 12272, *Bifidobacterium adolescentis* YIT 4011^T^, *Bifidobacterium bifidum* YIT 10347 and *Bifidobacterium longum* subspecies *longum* YIT 4021^T^ (hereafter called *B*. *longum*) in GAM broth (Nissui) supplemented with 1% (w/v) β-lactose were collected by centrifugation, washed twice and suspended in sterile PBS. An aliquot of the bacterial suspension was stained with DAPI for counting the number of bacteria by fluorescence microscopy. Bacteria suspensions were heat-killed by boiling for 30 minutes and heat-killed bacteria were then freeze-dried, suspended in PBS at a stock concentration of 5×10^10^/ml and kept in aliquots at -80°C.

### Statistics

All data shown represent the means +/- standard deviation. Statistical analyses were performed using two-sided Student’s t-tests with unequal variance, and statistically significant differences are indicated as follows: * p < 0,05; ** p < 0,01; *** p < 0,001.

## Results

### Tunicamycin-induced ER stress disrupts the barrier function of Caco-2 epithelial monolayers through plasma membrane rupture

**Fig 1:**
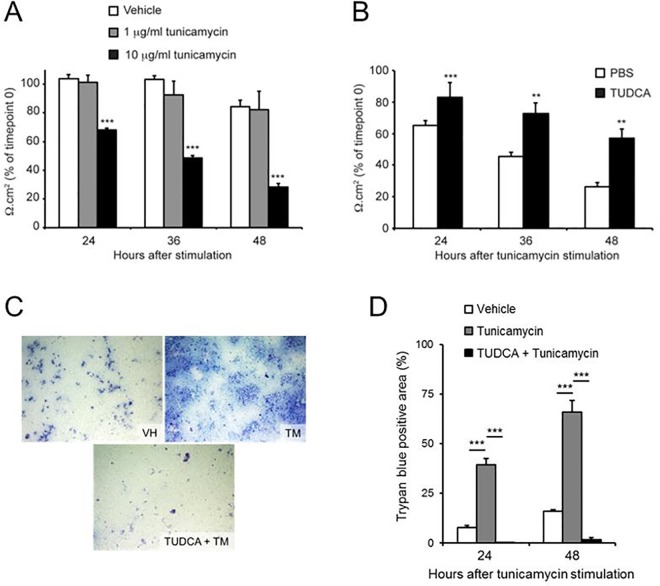
Tunicamycin-induced ER stress decreases epithelial barrier integrity in Caco-2 monolayers by plasma membrane disruption. Change in TEER of differentiated Caco-2 monolayers **(A)** after apical stimulation with vehicle or indicated concentrations of tunicamycin, **(B)** after one hour pre-treatment with PBS or TUDCA and subsequent apical stimulation with 10 μg/ml tunicamycin. **(C)** Representative images and **(D)** quantification of Trypan blue positive areas of differentiated Caco-2 monolayers stained with Trypan blue after one hour pre-treatment with PBS or TUDCA and subsequent apical stimulation with 10 μg/ml tunicamycin (TM) or vehicle (VH) for 48 hours. Results shown are the means ± standard deviation of three independent experiments, each using triplicate wells. Statistical analyses were performed using two-sided Student’s t-tests with unequal variance, with ** p < 0,01 and *** p < 0,001.

Maintenance of epithelial barrier function is essential for intestinal health. We therefore evaluated whether ER stress increases epithelial permeability of a monolayer of Caco-2 cells. For this purpose, different concentrations of the ER stress inducer tunicamycin (TM) were added to fully differentiated Caco-2 monolayers. While both vehicle treatment and 1 μg/ml TM did not cause a significant disruption of Caco-2 monolayer integrity during 48 hours of incubation, a concentration of 10 μg/ml of TM resulted in a gradual decrease of epithelial integrity to 32% of the initial TEER value after 48 hours (**Fig 1A**). In contrast, when Caco-2 monolayers were pre-treated with the ER stress inhibitor TUDCA, the TM-induced TEER decrease was significantly inhibited to 57% of the original TEER value after 48 hours (**Fig 1B**). These results show that treatment with 10 μg/ml TM causes Caco-2 epithelial barrier disruption by means of ER stress induction.

In order to evaluate the mechanisms underlying the decreased Caco-2 monolayer integrity caused by TM, we first investigated the effect of TM on the plasma membrane integrity (PMI) of the individual Caco-2 cells in the monolayer. Through staining Caco-2 monolayers with the cell-impermeable trypan blue dye, we observed a 5–6 fold increase of trypan blue-positive cells 24 and 48 hours after TM treatment when compared to control vehicle-treated Caco-2 monolayers (**Fig 1C and 1D**). In accordance with the above TEER observations, pre-treatment of Caco-2 monolayers with TUDCA strongly inhibited the appearance of such trypan blue-positive cells displaying PMI defects (**Fig 1C and 1D**). These results show that TM-induced epithelial barrier disruption was accompanied by PMI loss and suggest that PMI loss of individual cells contributes to ER stress induced-barrier decrease in differentiated Caco-2 monolayers.

### Lysosomal rupture contributes to ER stress-induced barrier loss in Caco-2 monolayers

**Fig 2:**
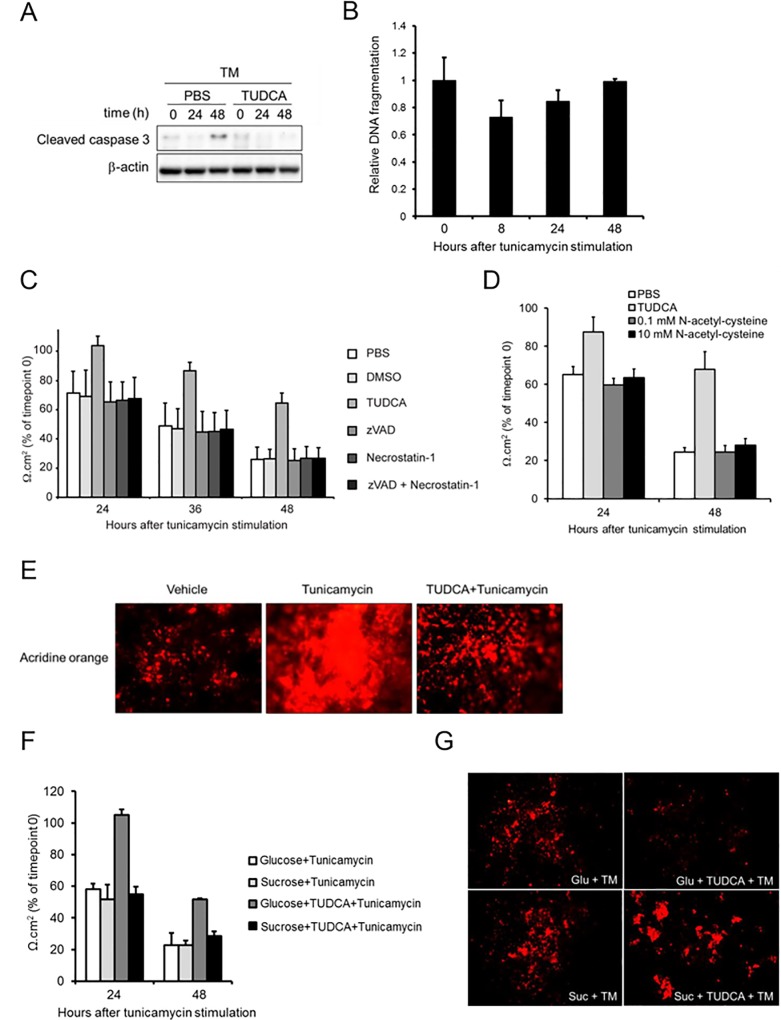
Tunicamycin disrupts Caco-2 monolayer integrity through caspase- and RIPK1-independent cell death that is associated with lysosomal membrane permeabilization. **(A)** Western Blot for active Caspase-3 in differentiated Caco-2 monolayers after apical stimulation of 10 μg/ml TM for indicated time periods with or without TUDCA pre-treatment. **(B)** Relative level of DNA fragmentation in differentiated Caco-2 monolayers after indicated time periods of apical stimulation with 10 μg/ml TM, Change in TEER of differentiated Caco-2 monolayers after one hour pre-treatment with **(C)** TUDCA, cell death inhibitor(s) (50 μM zVAD, 30 μM Necrostatin-1), **(D)** N-acetyl-cysteine (0.1 mM, 10 mM) or their vehicles (PBS, DMSO) and subsequent apical stimulation with 10 μg/ml tunicamycin during 48 hours. **(E)** Representative microscopic images of Caco-2 monolayers stained with acridine orange after one hour pre-treatment with TUDCA or PBS and subsequent apical stimulation with 10 μg/ml TM or vehicle for 48 hours. The experiment shown is a representative of two independent experiments. **(F)** Change in TEER and **(G)** Representative acridine stainings of differentiated Caco-2 monolayers after one hour pre-treatment with TUDCA or PBS together with glucose or sucrose and subsequent apical stimulation with 10 μg/ml tunicamycin or vehicle during 48 hours. The results shown are representative of two independent experiments and represent the means ± standard deviation of triplicate wells.

Because of the observed PMI loss in Caco-2 cells undergoing excessive ER stress, we analysed the involvement of cell death in TM-induced epithelial barrier disruption in Caco-2 monolayers. Remarkably, we observed the active form of Caspase-3, which is a hallmark of apoptosis, only 48 hours after TM treatment (**Fig 2A**). Even though TUDCA was able to prevent this event **(Fig 2A)**, such a late stage activation of Caspase-3 argued against caspase-dependent apoptotic cell death as the primary cause of TM-induced barrier loss in Caco-2 cells. In support of this hypothesis, no increase in caspase-dependent DNA fragmentation could be detected after TM treatment (**Fig 2B**), further arguing against a causative role for classical apoptotic cell death in TM-induced disruption of Caco-2 monolayers. Finally, treating Caco-2 monolayers with the pan-caspase inhibitor zVAD was not able to block TEER loss after TM treatment, showing that caspases are dispensable for this process (**Fig 2C**). Necroptosis is an alternative mode of cell death that often compensates for a cell’s inability to undergo caspase-dependent apoptosis.[20] Therefore, we treated Caco-2 monolayers also with a combination of zVAD and Necrostatin-1 (Nec1),[21] which inhibits RIPK1-dependent necroptotic cell death that has been implicated before in TM-induced cytotoxicity.[22] However, similarly to these individual inhibitors, also the combined actions of zVAD and Nec1 were not able to inhibit TM-induced Caco-2 barrier loss (**Fig 2C**). Together, these data showed that TM-induced barrier loss in Caco-2 monolayers is independent of both caspase-dependent apoptosis and RIPK1-dependent necroptosis. Furthermore, although antioxidants are known to diminish ER stress,[23] pre-treatment with the antioxidant N-acetyl-cysteine did not prevent TM-induced Caco-2 barrier loss (**Fig 2D**), thereby excluding also oxidative stress as a primary cause of this observation.

Given the observed loss of PMI after TM treatment, we further sought for the mechanisms underlying this event. Lysosome homeostasis is known to play a critical role in regulating PMI, as lysosomal membrane permeabilization (LMP) causes PMI after intracellular release of lysosomal acidic hydrolytic enzymes.[24] Strikingly, TM stimulation for 48 hours caused an accumulation of intracellular lysosomal contents, as the volume of acidic compartments (VAC) per cell probed by acridine orange was expanded in TM-treated Caco-2 monolayers. In contrast, this TM-induced phenomenon of VAC enlargement was much less apparent in TUDCA pre-treated and thus barrier-preserved Caco-2 monolayers (**Fig 2E**). These results thus suggested that ER stress provokes PMI loss in Caco-2 monolayers by lysosome disintegration. To further investigate this hypothesis, we treated Caco-2 monolayers with sucrose, which is known to cause lysosomal rupture.[25] Strikingly, in contrast to control glucose administration, addition of sucrose could override the barrier protective effect of TUDCA in Caco-2 monolayers after TM treatment (**Fig 2F and 2G**). Indeed, while TUDCA prevented VAC expansion and barrier disruption in glucose-treated Caco-2 monolayers, this barrier preserving effect of TUDCA was lost in sucrose-treated Caco-2 monolayers. This observation shows that an ER stress independent ability of sucrose to induce lysosomal rupture restores barrier loss of Caco-2 monolayers after TM treatment. Hence, lysosomal disintegration appears to be the critical TUDCA-sensitive mechanism by which TM-induced ER stress disrupts epithelial barrier integrity in Caco-2 monolayers.

### Bifidobacteria inhibit ER stress-induced epithelial barrier disruption in Caco-2 monolayer

**Fig 3:**
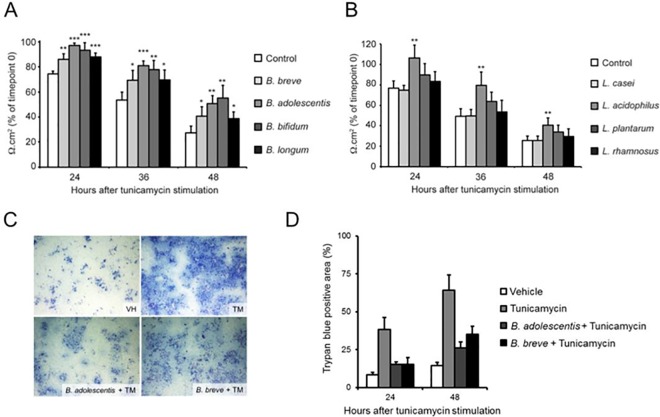
Bifidobacteria prevent tunicamycin-induced barrier loss in Caco-2 monolayers. **(A-B)** Change in TEER of differentiated Caco-2 monolayers after pre-treatment with indicated **(A)** bifidobacteria or **(B)** lactobacilli and subsequent apical stimulation with 10 μg/ml tunicamycin during 48 hours. Results shown are the mean ± standard deviation of three independent experiments each using triplicates. Statistical analyses were performed using two-sided Student’s t-tests with unequal variance, with * p < 0,05; ** p < 0,01 and *** p < 0,001. **(C-D)** Representative Trypan blue staining images **(C)** and quantification **(D)** of the trypan blue positive area of differentiated Caco-2 monolayers after bifidobacteria pre-treatment and subsequent apical stimulation with 10 μg/ml tunicamycin (TM) or vehicle (VH) for 48 hours. The results shown are representative of two independent experiments and represent the means ± standard deviation of triplicate wells.

After having established that ER stress induction results in loss of barrier integrity in Caco-2 monolayers, we wanted to test whether bacteria can interfere with this detrimental phenomenon. Interestingly, pre-treating epithelial Caco-2 monolayers with bifidobacteria displayed protective effects against ER stress induced barrier loss (**Fig 3A**). Among the strains tested, *B*. *adolescentis* and *B*. *bifidum* were the most potent inhibitors, as Caco-2 monolayers pre-treated with these strains retained 50% and 55% of their original TEER value, respectively, whereas control Caco-2 monolayers decreased to 27% of the original after 48 hours of TM treatment. In contrast to these bifidobacteria observations, pre-treatment of Caco-2 monolayers with lactobacilli did not result in a general preventive effect on TM-induced barrier loss (**Fig 3B**). Instead, among the *Lactobacillus* strains tested only *L*. *acidophilus* prevented TM induced barrier loss in Caco-2 monolayers. Whereas control Caco-2 monolayers retained only 25% of their initial TEER after 48 hours of TM treatment, *L*. *acidophilus* pre-treated Caco-2 cells still exhibited 41% of their starting TEER value after 48 hours of TM treatment (**Fig 3B**). In contrast, none of the other *Lactobacillus* strains tested displayed a statistically significant protective effect against apical ER stress induced barrier loss in Caco-2 monolayers. Importantly, in all the above TEER experiments, pre-treatment with bifidobacteria or *L*. *acidophilus* did not alter TEER values of vehicle treated Caco-2 cells (data not shown), arguing for an ER stress specific instead of a general barrier strengthening effect of these bacteria in Caco-2 monolayers. Thus, *B*. *adolescentis*, *B*. *bifidum*, *B*. *breve*, *B*. *longum* and *L*. *acidophilus* can inhibit apical ER stress induced loss of epithelial barrier integrity in Caco-2 cells. As we could previously show that ER stress induced barrier loss in Caco-2 cells is associated with PMI loss, we evaluated the effects of *B*. *adolescentis* and *B*. *breve*, two of the most potent bacteria capable of preserving the Caco-2 epithelial barrier, on this feature of barrier loss in Caco-2 monolayers. In accordance with their exhibited barrier protective effect in the above TEER experiments, *B*. *adolescentis* and *B*. *breve* limited the number of trypan blue-positive cells to 2–3 folds of the control value after 24 and 48 hours of TM treatment (**Fig 3C and 3D**), indicating that bifidobacteria prevent ER stress-induced Caco-2 barrier loss through PMI maintenance.

### Bifidobacteria inhibit ER stress-induced gene expression in Caco-2 monolayers

**Fig 4:**
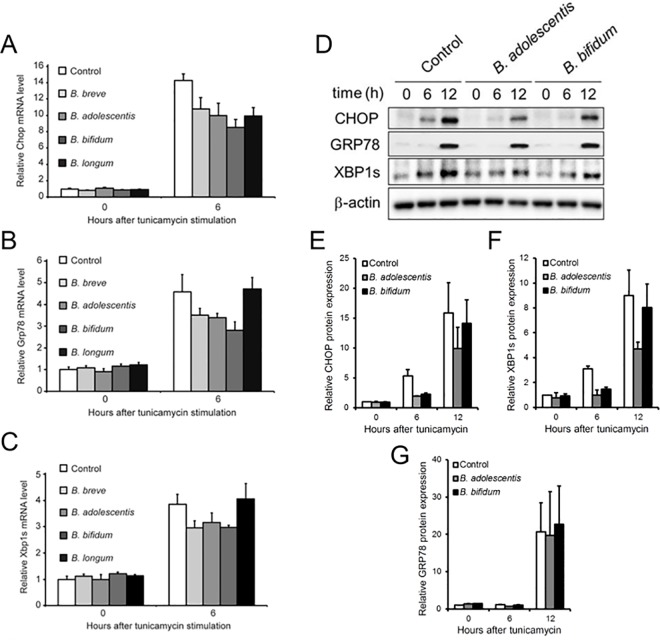
Bifidobacteria prevent tunicamycin-induced up-regulation of ER stress markers in Caco-2 monolayer. Relative mRNA expression of **(A)** Chop, **(B)** Grp78 and **(C)** XBP1s in differentiated Caco-2 monolayers after pre-treatment with indicated bacteria and subsequent apical stimulation with 10 μg/ml tunicamycin for 0 or 6 hours. All expression levels are relative to the expression level in non-treated control cells. The experiment shown is a representative of two independent experiments and the results displayed are the mean ± standard deviation of at least triplicate wells. **(D)** Protein expression of Chop, Grp78 and XBP1s in Caco-2 monolayers after pre-treatment with indicated bacteria and subsequent apical stimulation with 10 μg/ml tunicamycin for 0, 6 and 12 hours. Expression of β-Actin is shown as a loading control. The result shown is a representative of two independent experiments. **(E-G)** Quantification results of these two independent Western Blotting experiments, with all expression levels normalized to β-Actin and relative to the expression level in non-treated control cells. The quantifications shown are the means ± standard deviation of the two independent experiments.

The above results suggested that bifidobacteria inhibit apical ER stress induced loss of epithelial barrier integrity in Caco-2 monolayers in a manner similar to the chemical ER stress inhibitor TUDCA. Therefore, we investigated whether the barrier protective effect of bifidobacteria was associated with down-regulation of molecular features of ER stress. For this purpose, we evaluated the expression of the ER stress marker genes Chop, Grp78 and XBP1s that are typically up-regulated by UPR signalling upon ER stress induction.[3] As expected, the three UPR mRNAs were clearly induced in Caco-2 monolayer after 6 hours of TM treatment (**Fig 4A–4C**). In contrast, bifidobacteria showed remarkable inhibitory effects. This effect was most pronounced with Chop expression, which was suppressed by pre-treatment with each of the bifidobacterium strains (**Fig 4A**). Apart from *B*. *longum*, all bifidobacterium strains tested also inhibited expression of both Grp78 and XBP1s mRNAs at 6 hours after TM administration (**Fig 4B and 4C**). Moreover, in accordance with the mRNA expression results, *B*. *adolescentis* and *B*. *bifidum* showed clear inhibitory effects on Chop protein expression at 6 hours after TM treatment (**Fig 4D and 4E**). While these bacteria had an inhibitory effect also on XBP1s protein expression after 6 hours of TM (**Fig 4D–4F**), no impact on Grp78 protein expression could be detected (**Fig 4D–4G**). Taken together, these results indicate that except for *B*. *longum*, all bifidobacteria tested delay the expression of ER stress markers, suggesting that these strains are capable of inhibiting tunicamycin-induced ER stress in Caco-2 monolayers.

## Discussion

There are worldwide research efforts ongoing on the actions of probiotic bacteria, as these microorganisms are believed to confer intestinal health benefits to the host. However, although some studies suggested beneficial probiotic effects in IBD and IBS,[26, 27] their mechanisms of action remain poorly understood. Because the intestinal epithelium is the major contact site between luminal bacteria and the host, the presence of probiotics such as *Bifidobacterium* or *Lactobacillus* might directly influence IEC functions. In this respect, ER stress induced UPR was established as an important cellular stress response that acts in IECs to regulate intestinal homeostasis.[6] In this study, we illustrate the potentially detrimental role of excessive ER stress in a human IEC model, as barrier integrity of Caco-2 monolayers was severely compromised after ER stress induction by tunicamycin. In addition, we show observations suggesting that bifidobacteria as well as *L*. *acidophilus* possess beneficial ER stress inhibitory effects in this phenomenon. Importantly, ER stress inhibition was not a general feature of bacterial pre-treatment of Caco-2 monolayers, as a number of different lactobacilli were not able to inhibit ER stress and concomitant barrier loss in these IECs.

Although this observation suggests a specific interference of bifidobacteria with the ER stress-induced UPR, the underlying mechanisms are not fully clear yet. The UPR is initiated by three protein sensors at the ER membrane: inositol-requiring kinase 1 α (IRE-1α), pancreatic ER eIF2a kinase (PERK) and activating transcription factor 6 (ATF6). Accumulating misfolded proteins provoke each of these three sensor proteins to activate distinct UPR-associated transcription factors. The RNase activity of IRE1a mediates alternative splicing of Xbp1 leading to the production of the Xbp1s transcription factor, PERK-initiated phosphorylation of eukaryotic translation initiation factor 2α (eIF2α) enhances translation of the ATF4 transcription factor, and ATF6 is itself a transcription factor precursor that is activated through ER stress induced cleavage.[3] Our study showed that bifidobacteria have a selective effect in interfering with these three UPR signalling pathways. Indeed, bifidobacteria had no effect on ER stress induced production of Grp78, which is a target gene of the ATF6 UPR branch. Furthermore, although diminished Xbp1s production was observed after pre-treating Caco-2 cells with *B*. *adolescentis* and *B*. *bifidum*, this was not observed for *B*. *longum*. Given the shared capacity of these bacteria to prevent barrier loss after ER stress, this observation suggests that interfering with IRE1a-Xbp1 signalling is not a crucial asset for preserving epithelial integrity after ER stress. In contrast, all bifidobacteria strains that were found to impair TM-induced barrier loss also inhibited transcriptional upregulation of Chop, which is a target gene of the PERK-eIF2α-ATF4 branch of the UPR. This effect was confirmed on the protein level for *B*. *adolescentis* and *B*. *bifidum*, as these bacteria delayed Chop production after ER stress in Caco-2 monolayers. Collectively, our UPR signalling experiments suggest that a crucial ER stress inhibiting effect of bifidobacteria is situated in the PERK-eIF2α-ATF4 UPR branch. While the molecular basis remains to be identified, this observation suggests that bifidobacteria do not ameliorate ER stress through general upstream assistance in protein folding, but rather interfere downstream of protein misfolding in the PERK-eIF2α-ATF4 UPR pathway in order to prevent TM-induced barrier loss. Moreover, these results showing that specific interference in PERK-eIF2α-ATF4 signalling is associated with preventing barrier loss also suggest that PERK-eIF2α-ATF4-induced Chop expression could be a crucial mediator of epithelial injury. Indeed, mouse genetic data support this idea, as epithelial-specific transgenic expression of Chop made mice more susceptible to DSS-induced tissue damage.[17]

An interesting question relates to the mechanisms by which ER stress disrupts barrier integrity in Caco-2 monolayers. We showed that tunicamycin treatment of Caco-2 monolayers leads to loss of PMI, indicative of a cytotoxic effect of ER stress. Surprisingly however, we could show that TM-induced barrier loss occurs in a manner that is independent of caspase-mediated apoptosis as well as RIPK1-mediated necroptosis, two modes of cell death that have previously been implicated in TM-induced cytotoxicity in other cell types.[5, 22] Instead, we obtained evidence showing that TM-induced barrier loss in Caco-2 monolayers is associated with lysosomal membrane permeabilization leading to the release of lysosomal contents into the cytosol. Interestingly, also in leukaemia cells tunicamycin was shown to induce a lysosome rupture dependent cell death in which caspase-3 activation was a secondary phenomenon.[28] In addition, our tunicamycin observations are reminiscent of the resveratrol analogue pterostilbene, which induces a similar caspase-independent and RIPK1-independent cell death that relies on lysosomal membrane disruption.[29] Interestingly, differential susceptibilities of several human cell lines to this pterostilbene-induced cell death correlated with the expression level of Hsp70: the higher Hsp70 expression, the more resistant cells were to pterostilbene.[29] Because Hsp70 is a chaperone important in protein folding,[30] this suggests that also pterostilbene-induced cell death might be regulated by ER stress sensitivity. Together, these reports illustrate that the tunicamycin-induced LMP-associated cell death observed in Caco-2 cells may be a more general mode of ER stress cytotoxicity. However, the exact mechanisms by which TM-induced ER stress induces LMP and subsequent barrier loss in Caco-2 monolayers will require further investigation.

In conclusion, we established TM treatment of differentiated Caco-2 monolayers as an *in vitro* model for ER stress-induced loss of IEC barrier integrity, which supports a role for such an epithelial barrier disrupting effect of ER stress in the development of inflammatory intestinal diseases. We show that this detrimental effect of ER stress involves LMP and subsequent PMI loss that is independent of caspase-mediated apoptosis and RIPK1-dependent necroptosis. In addition, we provide evidence that bifidobacteria possess ER stress inhibitory capacities that can preserve the barrier integrity of Caco-2 monolayers after TM treatment. These results will trigger novel interest in the regulation of epithelial integrity by ER stress and the potential beneficial effects of bacteria in this process.
